# Effects of affective vs. instructional teacher scaffolding on preschoolers’ emotional engagement and social attention in picturebook reading

**DOI:** 10.3389/fpsyg.2026.1743106

**Published:** 2026-02-10

**Authors:** Sifan Wu, Tao Song

**Affiliations:** 1 Faculty of Innovation and Design, City University of Macau, Macao, Macao SAR, China; 2 School of Creative Arts and Design, Zhejiang Polytechnic University of Mechanical and Electrical Engineering, Hangzhou, China

**Keywords:** affective education, emotional engagement, eye-tracking, picturebook, social attention, teacher scaffolding

## Abstract

**Introduction:**

Emotional support is increasingly recognized as a critical component of effective early childhood education, yet empirical evidence comparing different scaffolding styles during classroom activities remains limited. This study examined how affective versus instructional teacher scaffolding shapes preschool children’s emotional engagement during electronic storybook reading.

**Methods:**

Forty-one children aged 4–6 years from one preschool were assigned by class to either an affective-scaffolding or instructional-scaffolding condition. Children’s regular classroom teachers, who received prior training, implemented the assigned scaffolding style while reading a 20-page digital picture book. Multimodal measures of engagement were collected, including eye-tracking data, children’s self-reports using the Self-Assessment Manikin (SAM), teacher ratings, and behavioral observations. Semi-structured interviews with children and teachers complemented the quantitative data.

**Results:**

Compared with instructional scaffolding, affective scaffolding led to significantly higher positive emotional experiences, reflected in greater emotional valence and arousal. Children in the affective condition also showed increased social attention, indexed by longer gaze duration on characters’ faces, and more frequent overt emotional engagement behaviors, without reduced attention to text. Engagement indicators were moderately intercorrelated, and qualitative findings converged with quantitative results, indicating higher perceived engagement under affective scaffolding.

**Discussion:**

The findings suggest that integrating emotional support into preschool reading activities can enhance children’s emotional involvement and focused participation. Affective scaffolding appears to be a trainable and assessable instructional strategy that promotes more holistic engagement in early childhood learning contexts.

## 1 Introduction

### 1.1 Research background and problem statement

Emotion education is a foundational component of early childhood learning because it supports children’s capacity to recognize, express, and regulate emotions, which in turn shapes classroom adjustment, peer interaction, and readiness to learn (Hudgins, 1979; Shearer et al., 2021). The preschool years constitute a sensitive period in which emotion-related competencies are rapidly developing and closely intertwined with emerging self-regulation and social understanding; therefore, emotionally supportive educational experiences can have lasting implications for children’s mental health and later social adaptation (Daniels, 2014). Importantly, emotion education is not an “add-on” to academic instruction. In early childhood classrooms, emotion-related cues embedded in everyday activities (e.g., storybook reading) can alter children’s moment-to-moment engagement by guiding attention, eliciting motivation to participate, and enabling empathic understanding. Yet, preschool practice often shows a “cognition-over-emotion” bias, in which teachers emphasize knowledge and skills while providing comparatively less support for children’s emotional experience and social development (Denham et al., 2012). This imbalance has motivated renewed attention to how classroom routines can simultaneously promote cognitive growth and emotional well-being, helping children develop as “whole learners” (Longobardi et al., 2022).

Picturebook reading is a pivotal activity in preschool education, valued not only for early literacy and language development but also as a natural vehicle for emotion education (Schoppmann et al., 2023). During joint reading of picturebooks, children actively construct story meaning based on their experiences, and effective teacher support is key to deeper understanding and engagement in this process (Neumann, 2020). Scaffolding theory, originating from Vygotsky’s concept of the zone of proximal development (ZPD), emphasizes that teachers provide calibrated support to help children progress beyond their current ability (van de Pol et al., 2010; Maryam et al., 2020). In shared book-reading contexts, teacher scaffolding can manifest not only as cognitive support (e.g., asking questions, explaining content) but also as emotional support (responding to and guiding children’s feelings) (Neumann, 2020; Deshmukh et al., 2022; Xiao et al., 2025). Practical experience indicates that when teachers highlight and guide the emotional aspects of a story and connect the story situation to children’s own experiences, it can heighten children’s sensitivity to emotional cues, build empathy, and spark positive parent–child interaction and emotional exchange (Dowdall et al., 2020; Schoppmann et al., 2023; Green and Sun, 2025). Thus, picturebook reading is a valuable opportunity to foster young children’s emotional development and social cognition, and its effectiveness largely depends on the type of interactive support teachers employ.

Accordingly, picturebook reading provides a practical and ecologically valid context for examining emotion education processes *in situ*: it is emotionally meaningful, socially interactive, and increasingly mediated by digital displays in contemporary classrooms. This context allows us to test whether emotionally oriented teacher scaffolding can systematically shift children’s subjective emotional experience, their allocation of visual attention to social–emotional cues in the narrative (e.g., characters’ faces), and their observable engagement behaviors—without compromising attention to textual information.

### 1.2 Theoretical framework and mechanisms

This study is grounded in sociocultural theory and complementary perspectives on attention and motivation to explain why teachers’ affective versus instructional scaffolding may differentially shape children’s engagement during picturebook reading.

#### 1.2.1 Sociocultural theory: scaffolding through emotionally meaningful cues

From a sociocultural perspective, learning and meaning-making are mediated through social interaction and culturally available semiotic tools. Scaffolding, rooted in Vygotsky’s ZPD, refers to the teacher’s contingent support that enables children to perform beyond their current independent capability (van de Pol et al., 2010; Maryam et al., 2020). In shared reading, teachers do not scaffold only through explicit questions or explanations; they also scaffold through paralinguistic and embodied cues—such as vocal prosody, facial expressions, gaze direction, gesture, and stance—that highlight what is emotionally relevant and guide children’s interpretation of the narrative (Deshmukh et al., 2022; Xiao et al., 2025). Such multimodal cues can function as “attention-directing” and “meaning-making” resources within the interaction, helping children notice characters’ emotional states and connect story events to felt experience (Kewalramani and Veresov, 2022). In this sense, affective scaffolding is conceptualized as an interactional form of scaffolding in which emotional cues are used to structure children’s participation and interpretation during reading, whereas instructional scaffolding relies primarily on cognitively oriented prompts and directives aimed at plot and text comprehension (Silliman et al., 2000; Pesco and Gagné, 2017).

#### 1.2.2 Attention and motivation: why affective scaffolding should increase social attention and engagement

Engagement in early childhood is commonly understood as multidimensional, involving emotional experience, behavioral participation, and attentional allocation (Frenzel et al., 2021). In classroom contexts, teacher emotional support—warmth, understanding, and encouragement—has been theorized to satisfy children’s relational needs and enhance their willingness to participate, thereby strengthening engagement (Alamos and Williford, 2020). Beyond global classroom climate, affectively rich teaching may operate through moment-to-moment attentional and motivational mechanisms. Positive affect has been linked to broader and more flexible cognitive processing, supporting sustained involvement in learning activities (Schoppmann et al., 2023; Wenren et al., 2024). Moreover, children show an early-developing sensitivity to faces and emotional signals; emotionally salient social cues can capture attention and promote social referencing processes (Peltola et al., 2018). During story reading, when a teacher uses emotionally expressive prosody and embodied cues, children may be more likely to allocate visual attention to characters’ faces and facial expressions to extract social–emotional information, whereas a comparatively neutral instructional style may not as strongly recruit this social-attentional pathway (Ruba and Pollak, 2020). Therefore, we expect affective scaffolding to (a) heighten children’s positive emotional experience, (b) increase social attention to characters’ faces, and (c) translate into more overt engagement behaviors, while not necessarily reducing attention to text.

### 1.3 Literature review and research gaps

Prior studies have highlighted the positive role of adult involvement in young children’s reading behaviors from various angles (van de Pol et al., 2010; Frenzel et al., 2021). Eye-tracking research shows that when an adult is present and actively co-reads with a child, the child’s overall attention level increases significantly, and they tend to fixate more on meaningful information and main characters in the story (Evans and Saint-Aubin, 2010). The parent’s presence and guidance help focus a child’s attention on key story information and prevent distraction (Guo and Feng, 2013). However, the specific modes of adult support can differ in orientation. Researchers have distinguished between cognitive scaffolding and emotional scaffolding as two interaction styles. For example, in parent–child reading contexts, parents’ use of comprehension questions and vocabulary assistance is categorized as cognitive scaffolding, whereas using praise, encouragement, and soothing to modulate the emotional climate is considered emotional scaffolding (Mackiewicz and Thompson, 2014). Survey findings indicate that parents often believe creating a joyful, intimate reading atmosphere is more important than providing cognitive stimulation alone, underscoring the value of emotional support in early reading experiences (Batini, 2020; Schapira and Grazzani, 2025). Evidence from classroom-based shared reading further suggests that teachers’ scaffolding behaviors are consequential for children’s participation and socio-emotional learning. In preschool settings, teachers commonly use prompts, elaborations, and follow-up moves during shared book reading, and such scaffolds can shape children’s talk and engagement with story content (Neumann, 2020; Deshmukh et al., 2022). Importantly, teachers also engage in emotion-focused talk and supportive responses that can promote children’s emotion understanding during book reading activities (Beazidou et al., 2013; Alamos and Williford, 2020). Therefore, in preschool settings, a teacher’s interactive style that balances both emotional and cognitive support may be more beneficial for children’s all-around development.

Despite growing research in related areas, several gaps remain for further exploration. First, there is a lack of direct comparative studies on the effects of affective vs. instructional scaffolding by teachers. Most existing literature either examines the general effectiveness of scaffolding or focuses on how digital media affect interaction patterns in parent–child reading, with few studies parsing the impact of different teacher guiding styles in real classrooms. Second, prior work has often relied on a single measurement channel (e.g., questionnaires, observation, or eye tracking), even though children’s engagement during shared reading is inherently multi-component. Visual attention indices capture real-time processing priorities (e.g., whether children attend to social–emotional cues such as faces), whereas teacher ratings summarize externally observable involvement across the session, and behavioral coding captures overt participatory acts that may signal emotional resonance and self-initiated interaction. Children’s own affective reports provide an indispensable subjective perspective that cannot be inferred from attention or behavior alone. Consequently, multi-source triangulation is needed to establish convergent validity across experiential, behavioral, and attentional layers of engagement and to avoid over-interpreting any single indicator as the engagement construct. This triangulation logic is consistent with research that (a) uses a combination of quantitative and qualitative methods (Yucelyigit, 2021), (b) collects interview data from teachers and parents in addition to interviews with children (Pecek and Zorec, 2022) and (c) uses eye-tracking as process data to reveal attention allocation during learning tasks (Ban et al., 2024). Finally, empirical research in the context of emotion education is still relatively sparse. Although theory suggests that emotionally supportive teaching can facilitate children’s emotional and social development, direct classroom experimental evidence is lacking. Particularly given the trend of digital picturebooks entering classrooms, it remains an open question how to effectively integrate emotional-education principles and guide teachers to use appropriate scaffolding strategies to enhance children’s engagement and emotional experience.

In light of the above analysis, this study conducted a classroom-based comparison of affective vs. instructional teacher scaffolding, using a combination of objective instrumentation and subjective assessments to fill the empirical gap regarding how affective interactions influence preschoolers’ social attention and engagement behaviors.

### 1.4 Research purpose and questions

This study aims to investigate the mechanisms by which teacher interaction style in an early childhood emotion education context affects children’s social attention and overt engagement behaviors, providing empirical guidance for improving preschool classroom practice. Specifically, we compare affective scaffolding (emphasizing emotional communication and support) with instructional scaffolding (emphasizing cognitive guidance) in a picturebook reading activity. We designed a classroom-based e-book reading task in which teachers interacted with children using either an affective or instructional style, and we collected data through multiple methods: eye-tracking to record children’s gaze distribution on three types of areas in the book (text, characters’ faces, and background), quantifying visual attention patterns; a child self-report emotional scale (the SAM pictorial scale), a teacher rating scale of child engagement, and video-based behavioral coding to build a comprehensive index system of children’s emotional and behavioral engagement. This multi-source, triangulated design aimed to capture children’s responses under different scaffolding conditions from multiple perspectives.

The study addressed the following quantitative research questions and *a priori* hypotheses:

RQ1a (Quantitative): Do children’s emotional experiences differ significantly between the affective scaffolding and instructional scaffolding conditions?

*H1a*: Children in the affective scaffolding condition will report higher emotional valence and higher arousal than children in the instructional scaffolding condition.

RQ1b (Qualitative): How do children describe their emotional experiences and perceived story meaning under affective versus instructional scaffolding?

RQ2a (Quantitative): Do teacher ratings of children’s emotional engagement differ by scaffolding type?

*H2a*: Teacher-rated emotional engagement will be higher in the affective scaffolding condition than in the instructional scaffolding condition.

RQ2b (Qualitative): How do teachers characterize their use of affective versus instructional cues during reading, and what differences do they perceive in children’s responses?

RQ3: Does children’s visual attention distribution during electronic storybook reading vary by scaffolding type?

*H3*: Affective scaffolding will increase the proportion of total fixation duration allocated to the Face AOI, while attention to the Text AOI will not be reduced relative to instructional scaffolding.

RQ4: Do children’s overt engagement behaviors differ by scaffolding type?

*H4*: Affective scaffolding will yield higher frequencies of positive engagement behaviors (e.g., pointing, emotion words, retelling, proximity) and a higher aggregated Behavioral Engagement Index (BEI) than instructional scaffolding, whereas re-reading requests and distraction will not differ significantly between groups.

RQ5: Are engagement indicators consistent across subjective experience, external ratings, attention, and behavior?

*H5*: Children’s valence and arousal, teacher-rated engagement, Face AOI attention proportion, and BEI will be positively correlated.

## 2 Materials and methods

### 2.1 Participants

Participants included two preschool teachers and 46 preschool children (ages 4–6 years, *M* = 5.2; 25 boys, 21 girls) recruited from a preschool in Macau. The two teachers were first recruited as participants and were assigned to implement either the affective scaffolding or instructional scaffolding condition. Randomization occurred at the classroom level, with intact class units (groups of children) assigned to each condition in order to avoid teacher cross-contamination all children were native Mandarin Chinese speakers with normal or corrected-to-normal vision and no reported developmental or neurological disorders. Because the intervention was delivered by teachers at the class level, children were assigned to conditions based on their classroom membership rather than individual randomization. After excluding data with insufficient eye-tracking quality, data from 41 children were retained for the final analyses. All children’s guardians provided written informed consent, and the children themselves gave verbal assent prior to participation. At the end of the study, all participants received a small gift as a token of appreciation. No performance assessments or penalties were involved at any stage. Demographic information for both teachers and children is presented in Table 1.

**Table 1:** Demographic information.

Variable		Affective group (*N* = 19)	Instructional group (*N* = 22)	Teacher (*N* = 2)
Age		5.16 (*SD* = 0.83)	4.95 (*SD* = 0.65)	31 (*SD* = 3.41)
Gender	Male	10 (52.6%)	13 (59.1%)	0 (0%)
	Female	9 (47.4%)	9 (40.9%)	2 (100%)
Education level	Bachelor’s degree	/	/	2 (100%)
Marital status	Married	/	/	2 (100%)
Teaching experience	Three years	/	/	2 (100%)

### 2.2 Research design

We employed a quasi-experimental mixed-methods design, with teacher interaction style as the independent variable. We compared preschool children’s visual attention and emotional engagement under two teaching conditions: affective scaffolding (emphasizing emotional communication and support) vs. instructional scaffolding (emphasizing cognitive instruction). A triangulated framework was used, combining eye-tracking, child emotional self-reports, teacher ratings, behavioral coding, and semi-structured interviews (Hartley and Sturm, 1997; Mao et al., 2026). The primary dependent measures included: (1) Visual attention indices – each child’s total fixation duration on three regions of the book (text, characters’ faces, and background), measured via eye-tracking (Total Fixation Duration, TFD for each AOI); (2) Emotional engagement indices – the child’s self-reported SAM ratings, the teacher’s emotional engagement rating scores, and frequencies of key behaviors from observation coding. Additionally, semi-structured interviews with children and teachers were conducted to gain deeper insight into children’s emotional experiences and the teachers’ strategies. The study followed APA ethical guidelines and received approval from the institutional ethics committee.

### 2.3 Stimuli

The stimulus was an original electronic picturebook titled *“Adventure Across the Sea,”* consisting of 20 pages of story content. It was displayed as high-definition images (1920 × 1,080 pixels) on a 24-inch LCD monitor (60 Hz refresh rate, ~200 cd/m^2^ brightness). The story follows a little duck who encounters a storm at sea and, through courage, cooperation with friends, and hope, eventually reaches a safe island. The narrative is infused with positive emotional themes such as “courage,” “friendship,” and “hope.” The picturebook’s visuals contain clear emotional cues (character facial expressions, actions, color changes, etc.), making it suitable for studying how children allocate attention and respond emotionally during an affect-rich reading experience.

**Figure 1:**
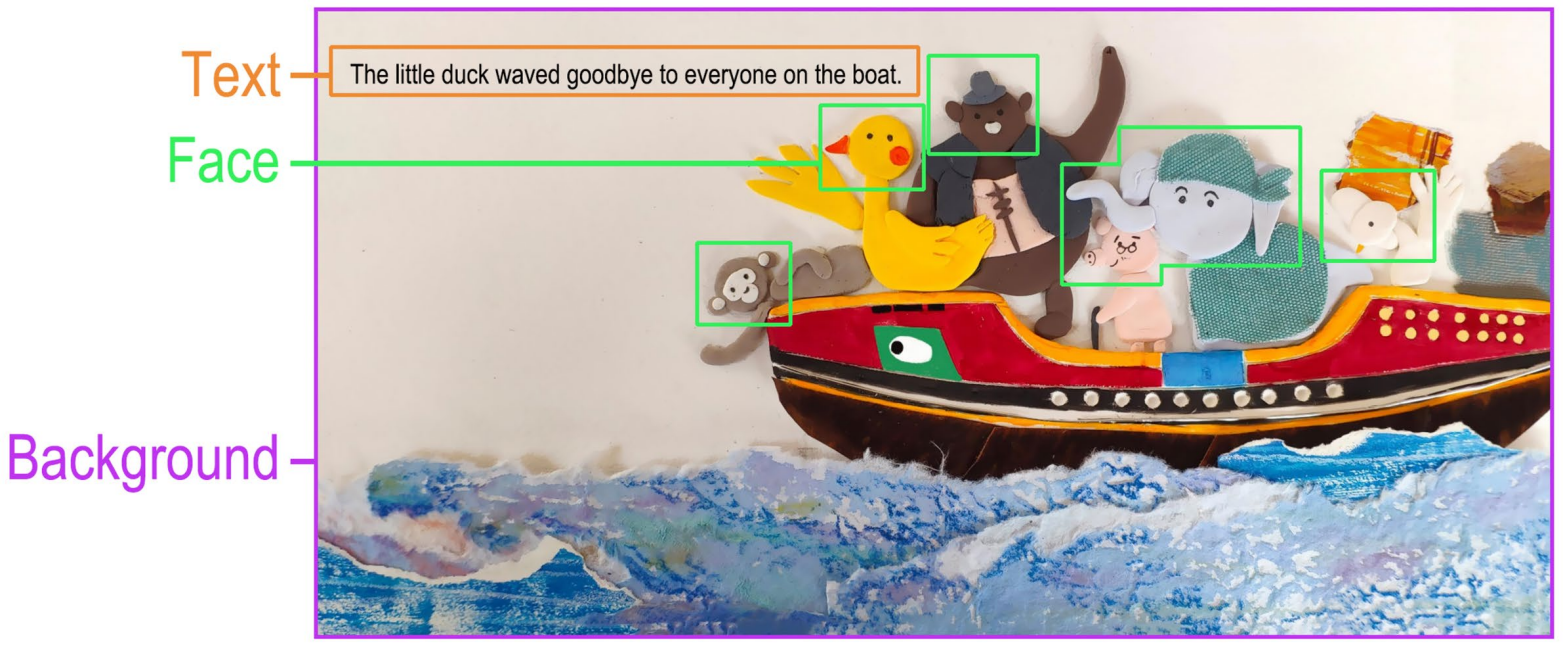
One page of the picturebook (marked with the AOIs classification).

Using Tobii Pro Lab software, we defined three AOIs on each page of the e-book (Figure 1): (1) Text AOI – all printed text; (2) Face AOI – characters’ faces and facial expression regions; (3) Background AOI – all other imagery besides text and faces (e.g., waves, boats, sky). The story was divided into three key narrative episodes (“Storm Strikes,” “Helping Each Other,” and “Reaching the Goal”) based on the plot progression, for analyzing differences in children’s emotional responses across story segments.

### 2.4 Apparatus and measures

Eye-movement data were collected using a Tobii Pro Fusion desktop eye tracker (Tobii Technology AB, Sweden) to capture children’s visual attention. This device uses infrared optical tracking at a sampling rate of 120 Hz, with spatial accuracy of ~0.5°. Each child sat alone in a quiet classroom area about 60 cm from the monitor. A standard 5-point calibration procedure was performed for each child using Tobii Pro Lab; any calibration error above 0.5° was recalibrated. Gaze data were processed in Tobii Pro Lab with the I-VT (Velocity-Threshold Identification) fixation filter (30°/s velocity threshold), excluding abnormal fixations shorter than 60 ms or longer than 1,000 ms. If a child’s overall tracking ratio fell below 80%, that dataset was excluded. This resulted in 41 valid eye-tracking datasets.

In addition to the eye tracker, the following instruments were used to assess children’s emotional engagement:

Self-Assessment Manikin (SAM) Child Pictorial Scale (5-point) – Used to measure each child’s emotional valence and arousal at the three key story episodes. The SAM is a cartoon figure depicting emotional states, ranging from “very unhappy” to “very happy” (for valence) and “very calm” to “very excited” (for arousal) on five-point scales (Bradley and Lang, 1994). At each episode pause, a research assistant stopped the story and verbally asked the child to point to the SAM figure that best represented the child’s feeling. The child’s valence and arousal scores were averaged across the three episodes to produce overall valence and arousal indices for that child.

Teacher Rating Scale of Child Engagement – After the reading session, the teacher rated each child’s emotional engagement during reading. The scale contains 5 items (e.g., “child displayed joyful emotion,” “child showed empathy with the story,” “child was focused,” “child expressed him/herself proactively,” “child showed emotional responses”), each rated on a 5-point Likert scale (1 = strongly disagree, 5 = strongly agree). The five items are summed to a total score (range 5–25), representing the level of emotional engagement observed by the teacher.

Behavioral Coding Checklist (Observer Checklist) – Two trained coders independently coded each child’s behavior from the session video. The coded behaviors included seven categories: pointing, emotion words, verbal recall/self-involvement, re-reading request, leaning toward the screen, and distraction. The first six are positive indicators of engagement, while “distraction” is a reverse indicator. A combined event sampling and interval sampling (10-s windows) method was used to tally occurrences (or duration for distraction) of each behavior. Results were recorded as frequency counts (or frequency per minute) for each behavior to standardize measures across children. Table 2 shows the behavioral coding scheme.

**Table 2:** Behavioral coding scheme (The Z1 to Z6 refer to six behaviors).

Behavior	Definition of count	Coding rule	Note
Z1 (Pointing)	Number of times the child points with finger or hand to a book character or emotional cue during reading.	Continuous pointing at the same element counts as 1; pointing to a new area counts as another.	Reflects visuo-motor coupling and attention to emotion cues.
Z2 (Emotion words)	Number of times the child utters an emotionally valenced word or phrase (e.g., “scary,” “so happy”).	Repeated synonyms not double-counted; separate occurrences in same scene count individually.	Reflects emotional expression and empathic understanding.
Z3 (Verbal recall)	Number of times the child recounts story content or speaks in first-person as if taking part (“I…” “we…”).	Each complete statement counts as 1.	Reflects narrative comprehension and emotional identification.
Z4 (Re-reading request)	Number of times the child proactively requests to view or read a page/section again.	Explicit requests (e.g., “I want to see that again”) count each time.	Indicates active emotional engagement in content.
Z5 (Leaning forward)	Number of times the child’s body noticeably leans forward toward the screen or table.	Each instance of leaning lasting >2 s counts as 1.	Indicates concentration and involvement.
Z6 (Distraction)	Number of times the child’s gaze or attention clearly leaves the book (looking away, side talking, etc.).	Each instance >2 s counts as 1; this measure is later reverse-scored.	Reflects attention lapse (reverse indicator).

Semi-Structured Interview Guides: After the reading activity, the researcher conducted 5–8 min interviews separately with a subset of children and with the teachers to probe emotional experiences and teaching strategies. Both the student and teacher interview guides each consisted of eight questions (see Appendix). Example child interview questions included: “Which page did you like the most or find the scariest? Why?,” “How do you think the little duck felt on that page?,” “If you were on the boat, what would you do?” Teacher interview questions included: “How did you use language or tone to help children understand emotions?,” “During which parts of the story did the children seem most engaged, and how did you respond?,” “In your opinion, what are the differences between the affective and instructional guiding approaches?” All interviews were audio-recorded with permission and transcribed for thematic analysis.

### 2.5 Procedures

The experiment consisted of three phases: teacher training, the reading experiment, and a post-reading assessment.

Training Phase: Each of the two teachers received training on an interaction script corresponding to their assigned scaffolding style. The training was led by the research team and consisted of four centralized training sessions (two instructional sessions and two practice sessions), each lasting approximately 1.5 h. The training covered the principles and demonstrations of scaffolding strategies specific to each condition, ensuring that the teachers fully understood and could consistently implement the corresponding intervention approaches. The teacher in the affective scaffolding condition was trained to use emotional language, empathetic questioning, and warm tone (e.g., asking “Do you think the little duck is scared here?” or noting “It is so brave, is not it?”), whereas the teacher in the instructional scaffolding condition was trained to use a neutral tone and cognitive questions (e.g., “Who is this?” “What is the duck doing now?”). This was to ensure a clear contrast in the emotional content of the two scaffolding styles. Research assistants provided video demonstrations and monitored practice sessions to ensure consistency in implementation.

Reading Session: Each child sat facing the monitor to view the electronic picturebook. The teacher sat to the child’s right and provided verbal guidance according to the assigned style. The reading session lasted no more than 10 min. Children could turn pages on their own or ask the teacher to help with page-turning. The Tobii Pro Fusion eye tracker recorded the child’s gaze data throughout. At the three predetermined story episodes, the session was paused, and the research assistant guided the child to complete an immediate SAM rating of their current emotion. After finishing the story, the teacher immediately filled out the engagement rating scale for that child. The entire session was video-recorded for subsequent behavioral coding. For each condition, 6 children were randomly selected for the post-reading interview, and both participating teachers were interviewed. To ensure fidelity of implementation, all reading sessions were monitored and video-recorded. Two trained researchers independently reviewed the recordings using a predefined checklist. Implementation fidelity was verified using a structured checklist focusing on key indicators of affective versus instructional scaffolding (Appendix). Two trained researchers independently reviewed video recordings of each session, and all sessions met the predefined fidelity criteria. Sessions were judged as meeting fidelity criteria only if the assigned scaffolding style was consistently maintained throughout the reading. Inter-rater agreement was high, and no sessions were excluded due to insufficient fidelity. These procedures provide verifiable evidence that the two scaffolding conditions were implemented as intended.

Post-Reading Assessment: In addition to the teacher’s rating and interviews mentioned above, all collected data (eye-tracking, SAM, behavior counts) were compiled for analysis.

### 2.6 Data analysis

All eye-tracking data, emotional engagement measures, and behavioral coding data were analyzed using IBM SPSS Statistics 26.0.

Eye-tracking data from Tobii Pro Fusion were first preprocessed by excluding any sample with <80% gaze tracking and removing anomalous fixations (<60 ms or >1,000 ms). We then exported each child’s total fixation duration on the text, face, and background AOIs from Tobii Pro Lab, and calculated the proportion of fixation time on each AOI relative to the total fixation duration. These proportional measures represent the child’s visual attention distribution across different elements of the picturebook.

Children’s emotional engagement was assessed via three sources: SAM self-ratings (valence and arousal), the teacher rating scale, and a behavioral emotional engagement index (BEI) derived from observational data. The SAM valence and arousal scores were averaged across the three key story episodes to yield overall valence and arousal scores. The average of the five items on the teacher rating form represents the level of children’s emotional involvement observed by the teacher.

Behavioral data consisted of the six categories of overt behavior during reading: pointing, emotion words, story retelling, re-reading requests, leaning forward, and distraction. All behaviors were standardized to frequency per minute to account for session length. Distraction was treated as a reverse indicator. To combine different behaviors on a common scale, we standardized the frequency of each behavior (z-score within the sample):


$$Z_{i}=\frac{\mathrm{Xi}-\bar{X}i}{SD_{i}}\;$$

Where $Z_{i}$ represents the standardized score of the *i*-th behavior, and ${\bar{X}}_{i}$ and $SD_{i}$ denote the mean and standard deviation of that behavior across the full sample, respectively. The distraction behavior was reverse-coded after standardization (i.e., multiplied by −1).

Subsequently, the Behavioral Engagement Index (BEI) was calculated using equal-weighted aggregation across all behaviors:


$$\mathrm{BEI}=\frac{1}{6}(Z_{1}+Z_{2}+Z_{3}+Z_{4}+Z_{5}-Z_{6})$$

A higher BEI indicates stronger overall observable emotional engagement by the child during reading.

For descriptive statistics, we computed each variable’s mean (*M*) and standard deviation (*SD*). To address RQ1a, we conducted independent-samples t tests to compare children’s SAM valence and arousal between the affective and instructional scaffolding groups; to address RQ1b, we transcribed children’s post-activity interviews verbatim and analyzed them thematically to capture how they described their emotional experiences and perceived story meaning under each condition. To address RQ2a, we used independent-samples t tests to compare teacher-rated emotional engagement between groups; to address RQ2b, we transcribed teacher interviews and applied thematic analysis to characterize teachers’ affective versus instructional cues during reading and their perceptions of children’s responses. To address RQ3, we conducted independent-samples t tests on eye-tracking outcomes, specifically the proportion of total fixation duration allocated to the Face, Text, and Background AOIs. To address RQ4, we conducted independent-samples t tests to compare the frequencies of coded engagement behaviors and the aggregated Behavioral Engagement Index (BEI) across conditions. For all t tests, Levene’s test was examined to assess homogeneity of variance; when Levene’s test indicated unequal variances (*p* < 0.05), Welch’s corrected t test was reported. All tests were two-tailed with *α* = 0.05. Effect sizes were calculated using Cohen’s *d* to quantify the magnitude of group differences. Given the small sample size, we conducted a sensitivity analysis to assess the statistical power. Under the conditions of a two-tailed α = 0.05 and 80% statistical power, the minimum effect size detectable in this study (n₁ = 19, n₂ = 22) was Cohen’s d = 0.91. To address RQ5, we computed Pearson correlations among SAM valence, SAM arousal, teacher-rated engagement, Face AOI fixation proportion, and BEI to examine convergence across subjective experience, external ratings, attention, and behavior. Finally, qualitative themes were integrated with quantitative results at the interpretation stage to contextualize group differences and support triangulation (e.g., linking higher Face AOI attention and BEI in the affective condition to interview accounts of emotion talk, empathic meaning-making, and self-initiated participation).

## 3 Results

### 3.1 Subjective emotional ratings

Descriptive statistics and *t*-test results for children’s self-reported emotional valence and arousal are shown in Table 3. The affective scaffolding group had significantly higher overall emotional valence (*M* = 3.58, *SD* = 0.38) than the instructional scaffolding group (*M* = 3.24, *SD* = 0.47), *t* (39) = 2.476, *p* = 0.018. Similarly, the affective group’s overall arousal level (*M* = 4.07, *SD* = 0.45) was significantly higher than the instructional group’s (*M* = 3.50, *SD* = 0.48), *t* (39) = 3.892, *p* < 0.001.

**Table 3:** Descriptive statistics and group comparisons for children’s emotional valence and arousal (Self-Assessment Manikin ratings).

Emotional measure	Group	*N*	*M*	*SD*	*t*	*df*	*p*	*Cohen’s d*
Valence (Episode 1)	Affective	19	2.21	0.92	−0.358	39	0.722	−0.11
	Instructional	22	2.32	0.99				
Arousal (Episode 1)	Affective	19	4.00	0.75	2.254	39	0.030	0.71
	Instructional	22	3.41	0.91				
Valence (Episode 2)	Affective	19	4.21	0.79	2.238	39	0.031	0.70
	Instructional	22	3.59	0.96				
Arousal (Episode 2)	Affective	19	4.11	0.94	2.230	39	0.032	0.69
	Instructional	22	3.50	0.8				
Valence (Episode 3)	Affective	19	4.32	0.67	2.146	39	0.038	0.67
	Instructional	22	3.82	0.80				
Arousal (Episode 3)	Affective	19	4.11	0.74	2.480	39	0.018	0.77
	Instructional	22	3.59	0.59				
Overall Valence	Affective	19	3.58	0.38	2.476	39	0.018	0.77
	Instructional	22	3.24	0.47				
Overall Arousal	Affective	19	4.07	0.45	3.892	39	< 0.001	1.22
	Instructional	22	3.50	0.48				

Looking at the three key story episodes individually, on the “Helping Each Other” and “Reaching the Goal” segments, children in the affective scaffolding condition reported significantly higher valence and arousal than those in the instructional condition (*p* < 0.05 for all comparisons). In the “Storm Strikes” segment, the affective group’s arousal was also significantly higher, *t* (39) = 2.254, *p* = 0.030, whereas valence did not differ significantly between groups for that segment, *t* (39) = −0.358, *p* = 0.722.

### 3.2 Teacher ratings of engagement

Descriptive and *t*-test results for the teacher’s observational engagement ratings are presented in Table 4. The affective scaffolding group’s teacher-rated engagement scores were higher (*M* = 3.91, *SD* = 0.76) than the instructional group’s (*M* = 3.38, *SD* = 0.66), and this difference was statistically significant, *t* (39) = 2.356, *p* = 0.024. In other words, teachers observed more intense emotional involvement from children in the affective condition compared to the instructional condition.

**Table 4:** Descriptive statistics and *t*-test for teacher-rated emotional engagement.

Group	*N*	*M*	*SD*	*t*	*df*	*p*	*Cohen’s d*
Affective scaffolding	19	3.91	0.76	2.356	39	0.024	0.74
Instructional scaffolding	22	3.38	0.66		39		

### 3.3 Visual attention distribution

The proportions of children’s fixation durations on the face, text, and background areas, along with the results of the *t*-tests, are presented in Table 5. The results show that for the face area, children in the affective scaffolding group spent a significantly higher proportion of fixation time (*M* = 0.26, *SD* = 0.04) compared to those in the instructional scaffolding group (*M* = 0.15, *SD* = 0.03), *t* (39) = 9.484, *p* < 0.001. For the background area, the fixation proportion was significantly lower in the affective group (*M* = 0.66, *SD* = 0.04) than in the instructional group (*M* = 0.77, *SD* = 0.04), *t* (39) = −9.501, *p* < 0.001. In contrast, for the text area, no significant difference was found between the two groups (*p* = 0.430).

**Table 5:** Proportion of total fixation duration on each area of interest (AOI) by group.

AOI	Group	*N*	*M*	*SD*	*t*	*df*	*p*	*Cohen’s d*
Face	Affective	19	0.26	0.04	9.484	39	< 0.001	2.96
	Instructional	22	0.15	0.03				
Text	Affective	19	0.09	0.04	0.798	39	0.430	0.25
	Instructional	22	0.08	0.04				
Background	Affective	19	0.66	0.04	−9.501	39	< 0.001	−2.99
	Instructional	22	0.77	0.04				

### 3.4 Behavioral observations

Descriptive statistics and *t*-test results for children’s behavior coding during reading are presented in Table 6. The findings indicate that the Behavioral Engagement Index (BEI) was significantly higher in the affective scaffolding group (*M* = 0.38, *SD* = 0.31) compared to the instructional scaffolding group (*M* = −0.33, *SD* = 0.37), *t* (39) = 6.619, *p* < 0.001. Among the six specific behaviors coded, four behaviors—pointing, emotion words, verbal recall, and leaning forward—showed significant group differences (*p* < 0.05), with the affective group consistently scoring higher than the instructional group. For the other two behaviors—re-reading requests and distraction—no significant differences were found between groups (*p* > 0.05).

**Table 6:** Children’s behavioral engagement during reading: descriptive stats and group comparisons.

Behavior	Group	*M (per min)*	*SD*	*t*	*df*	*p*	*Cohen’s d*
Pointing	Affective	2.13	0.49	6.727	30.831	< 0.001	2.23
	Instructional	1.24	0.33				
Emotion words	Affective	6.64	3.63	2.724	39	0.010	0.84
	Instructional	4.01	2.50				
Verbal recall	Affective	2.68	0.99	2.796	39	0.008	0.87
	Instructional	1.83	0.96				
Re-reading requests	Affective	0.83	0.31	−0.877	39	0.386	0.28
	Instructional	0.96	0.58				
Leaning forward	Affective	1.41	0.72	3.603	21.340	< 0.001	1.14
	Instructional	0.78	0.24				
Distraction	Affective	0.71	0.21	−1.468	30.414	0.170	−0.46
	Instructional	0.87	0.46				
BEI	Affective	0.38	0.31	6.619	39	< 0.001	2.04
	Instructional	−0.33	0.37				

### 3.5 Correlation analysis

Pearson correlation analyses were conducted among emotional valence, arousal level, teacher ratings, face fixation proportion, and BEI, with results presented in Table 7. The results showed a significant positive correlation between emotional valence and arousal, *r* = 0.794, *p* < 0.001. The proportion of fixation on faces was significantly positively correlated with emotional valence (*r* = 0.501, *p* = 0.001), arousal (*r* = 0.580, *p* < 0.001), teacher ratings (*r* = 0.479, *p* = 0.002), and BEI (*r* = 0.543, *p* < 0.001). In addition, teacher ratings were significantly positively correlated with BEI (*r* = 0.460, *p* = 0.002).

**Table 7:** Results of correlation analysis.

Variable	Correlations	Valence	Arousal	Teacher rating	Face attention	BEI
Valence	r	\	\	\	\	\
Arousal	r	0.794**	\	\	\	\
Teacher rating	r	0.228	0.279	\	\	\
Face attention	r	0.501*	0.580**	0.479*	\	\
BEI	r	0.259	0.284	0.460*	0.543**	\

*p < 0.05, **p < 0.001.

### 3.6 Qualitative findings

Through thematic analysis of the child and teacher interview transcripts, three major themes were identified, reflecting typical differences between the affective-scaffolding and instructional-scaffolding groups in children’s emotional experiences and interactive behaviors during picturebook reading. For clarity, “C-A#” denotes a child from the affective-scaffolding group, “T-A” the teacher in the affective condition; “C-B#” denotes a child from the instructional-scaffolding group, and “T-B” the teacher in the instructional condition. Each theme is illustrated with representative verbatim quotations.

Theme 1: Affective resonance and pleasurable experience. Under affective scaffolding, children exhibited stronger emotional resonance and more positive affective experiences. Several children in the affective group spontaneously described their own emotional reactions and their feelings toward the characters. For example, recalling the “storm” episode, one child said, “When the big waves came, I felt a little nervous. The little duck must have been very scared, but later it was very brave and got through it” (C-A3). This child not only recognized the little duck’s fear but also articulated a shift in his own feelings from tension to relief as the plot unfolded. Another child especially enjoyed the story’s ending: “My favorite page is when the little duck and its friends arrive at the destination, because everyone is happy and I felt happy too” (C-A5). These accounts suggest that affective scaffolding fostered children’s resonance with the story’s positive emotions and yielded pronounced pleasure. By contrast, children in the instructional-scaffolding group were less likely to spontaneously mention their own emotions and were more inclined to offer objective plot descriptions. When asked about the storm scene, one child responded, “The little duck should be scared there because the waves are big. I think it’s okay; I’m not very scared” (C-B4). The child recognized the character’s likely emotion but expressed his own feelings rather flatly. Teacher observations in the instructional condition echoed this difference: “Children can answer my questions, but they rarely take the initiative to talk about the characters’ emotions or their own feelings” (T-B). Overall, affective scaffolding prompted stronger emotional resonance and more explicit positive affective engagement, whereas children’s emotion expressions under instructional scaffolding were comparatively subdued.

Theme 2: Active participation and interactive behaviors. Children in the affective-scaffolding group displayed more self-initiated interactive behaviors during reading, indicating heightened focus and engagement. Teacher T-A noted: “Some children could not help but echo the dialogue in the story or point at the pictures—these spontaneous behaviors show they were truly engaged” (T-A). For instance, one child recalled the post-challenge scene: “I saw the little duck and its friends talking and hugging, and I pointed it out to the teacher because that moment felt very moving to me” (C-A2). The child’s spontaneous pointing and affective expression indicate deep immersion in the story context. Children in the affective group also sometimes imitated the story’s language or repeated characters’ lines, which deepened their sense of participation. In contrast, under instructional scaffolding, children’s participation was more often reactive to teacher prompts. As one child admitted, “The teacher asked me on every page ‘Who is this? What happened?’ and I just answered… I did not point to the pictures on my own” (C-B1). Thus, interaction in the instructional group centered on answering questions, with fewer spontaneous pointing or echo-reading behaviors. Notably, in both conditions, some children showed strong interest in particularly exciting scenes and occasionally requested re-reading favorite pages—for example, the “big wave” scene to re-experience the excitement, or the “mutual help” scene to revisit a warm moment. Such behaviors appeared in both groups without clear differences, reflecting high interest and engagement across conditions. Both teachers also reported that most children maintained attention during reading, with only occasional lapses and no obvious between-group difference. Overall, affective scaffolding encouraged more self-initiated verbal and bodily participation in story interaction, whereas participation under instructional scaffolding was more often guided by teacher questioning.

Theme 3: Teacher guidance style and children’s responses. The teachers’ interactive styles under different scaffolding approaches directly shaped children’s emotional expression and engagement states. The affective teacher employed a warm, empathetic guiding strategy that created an open and safe climate. As T-A explained: “I intentionally used emotional language and a gentle tone—asking things like ‘Is the little duck scared here?’—to help children understand emotions and to encourage them to share their feelings” (T-A). Such emotionally caring questions and tone made children more willing to disclose inner experiences. Children were also sensitive to the teacher’s affective style. One child noted: “When Teacher A told the story, her voice was gentle, and she would act out the little duck being sad and happy. I thought it was fun, and I felt nervous and happy along with the story” (C-A4). The teacher’s affective investment and lively performance heightened the child’s sense of immersion, synchronizing the child’s emotions with the narrative. By contrast, the instructional teacher tended to use a neutral tone and knowledge-focused prompts. As T-B put it: “I mostly asked in a neutral tone—‘Who is this? What happened?’—and the children could answer, but their emotional expressions were relatively fewer. Compared with this, I feel affective guidance might draw out more of children’s emotional responses” (T-B). Under such guidance, children concentrated more on identifying and remembering plot details and were less likely to express personal feelings spontaneously; some appeared reserved, focusing on answering rather than sharing affect. In sum, the affective teacher’s empathetic communication effectively stimulated children’s emotional participation and enthusiasm for interaction, whereas the instructional teacher’s strategy ensured plot comprehension and attention but elicited comparatively muted emotional responses. These qualitative patterns converge with the quantitative results: children in the affective group received higher teacher ratings and higher behavioral engagement indices, while children in the instructional group completed comprehension tasks but showed somewhat lower affective investment. Together, the themes and quotes corroborate the quantitative trends, revealing from multiple angles the positive impact of affective scaffolding on children’s emotional engagement and interactive behavior in the picturebook-reading context.

## 4 Discussion

In this study, we compared affective versus instructional teacher scaffolding during preschool electronic storybook reading using a mixed-methods design. Across children’s self-reported emotion (SAM), teacher ratings, eye-tracking indices (AOI gaze proportions), and observed behaviors (including BEI), the results converged on a clear pattern: relative to instructional scaffolding, affective scaffolding was associated with more positive and activated emotional experience, higher teacher-rated emotional engagement, and greater attention to characters’ faces without reduced attention to text. Qualitative interviews with children and teachers were consistent with these quantitative trends and helped clarify how emotionally responsive prompts and interactional norms may make emotions more salient and shareable during shared reading. The following sections interpret these findings in relation to relevant theory and prior empirical work and discuss implications for emotion-focused classroom practice.

### 4.1 Differences in children’s emotional experience

Our first major finding is that affective scaffolding significantly enhanced children’s emotional valence (positivity) and arousal during the learning activity. In other words, compared to instructional scaffolding, children who received emotional support from the teacher experienced more positive and more intense emotions. This finding resonates with educational psychology’s understanding of positive emotions: when young children feel interest, enjoyment, and other positive emotions in class, it aids their attention, self-regulation, and memory (Park, 2016). Prior research shows that integrating emotional support into scaffolding can stimulate children’s intrinsic motivation and willingness to engage (Wenren et al., 2024). Affective scaffolding creates a warm, empathetic learning atmosphere; through emotional contagion and positive feedback, the teacher causes children to feel greater interest and pleasure toward the learning material (Saarinen, 2020).

From a sociocultural perspective, affective scaffolding can be understood as interactional support within children’s ZPD, where the teacher’s empathic prompts and emotionally meaningful language function as mediational tools that help children make sense of the story’s emotional meanings in a shared activity (van de Pol et al., 2010; Renshaw, 2013) By legitimizing feelings as ‘discussable content’ during reading, the teacher may reduce children’s affective uncertainty and increase psychological safety, thereby facilitating more positive and activated emotional states (Morcom, 2014; Park, 2016).

The qualitative findings of the present study directly complemented the quantitative results, which showed that affective scaffolding led to significantly higher emotional valence and arousal. Children in the affective condition not only reported more positive emotions in the quantitative measures, but also articulated these emotions more explicitly in interviews, describing feelings of happiness, interest, and empathy, as well as displaying observable excitement and involvement. These qualitative accounts converge with the quantitative findings, suggesting that affective scaffolding not only elevates children’s reported emotional states but also renders those emotions more salient and shareable within classroom interaction. These patterns are consistent with prior qualitative evidence reported by (Beazidou et al., 2013), who found that children receiving emotional support were more likely to express positive emotions and empathy through spontaneous verbalizations and visible involvement. Such positive emotional experience is not merely about children “liking” the activity; it is also likely to facilitate subsequent cognitive processing and learning (Altmann et al., 2012; Briesemeister et al., 2015). Consistent with broaden-and-build accounts of positive emotion, heightened positive affect may broaden children’s moment-to-moment attentional readiness to explore narrative cues and sustain engagement across episodes, offering a plausible pathway from affective support to downstream learning involvement (Altmann et al., 2012; Briesemeister et al., 2015). In summary, regarding RQ1, our study demonstrates that affective scaffolding effectively increased children’s valence and arousal levels during a learning task, providing empirical support for the importance of emotion in teaching.

### 4.2 Differences in teacher-rated emotional engagement

Our second finding is that teacher-rated child engagement differed by condition: children in the affective scaffolding group received significantly higher engagement scores from the teacher than those in the instructional group. This means that in classrooms with affective scaffolding, the teacher observed children displaying more involved and enthusiastic emotional behaviors (e.g., happier facial expressions, more excited demeanor). This result aligns with prior research: when teachers provide emotional support, it often increases students’ engagement and motivation to learn (Guo et al., 2025). Although much of the engagement literature has focused on older students, teacher warmth and responsiveness appear to be particularly important in early childhood classrooms, where emotion regulation and participatory behaviors are closely co-developed (Alamos and Williford, 2020; Pakarinen et al., 2020). Prior findings suggest that an atmosphere of care, understanding, and encouragement from the teacher can elicit more positive emotional responses and greater participatory behavior among young children (Elam et al., 2010; Beazidou et al., 2013; Ho, 2025). In our study, teachers’ more enthusiastic affect and empathetic responses in the affective condition were associated with higher levels of emotional engagement among the children. This pattern is consistent with Skinner and Belmont’s classic work showing that when teachers demonstrate involvement and warmth, students are more likely to engage positively in class and display more on-task behavior (Skinner and Belmont, 1993). From a motivation-based perspective, affective scaffolding likely supports children’s relational needs (e.g., feeling understood and encouraged), thereby increasing approach-oriented participation and making engagement more observable to the teacher in real time (Pakarinen et al., 2020; Frenzel et al., 2021). In sociocultural terms, the teacher’s affective stance can be viewed as shaping the interactional norms of the shared reading activity—children are invited to contribute not only “correct answers” but also feelings and interpretations, which can increase visible engagement behaviors during classroom discourse (Renshaw, 2013).

In our research, the higher teacher ratings under the affective condition reflect that children were indeed more emotionally engaged, potentially creating a virtuous cycle: the children’s positive emotional feedback also heightened the teacher’s instructional enthusiasm (Maryam et al., 2020; Alavi and Esmaeilifard, 2021). Teacher interview feedback likewise supported this point—the teacher in the affective condition reported that children “had a sparkle in their eyes” and “participated in discussion with great eagerness,” indicating a higher level of emotional engagement. These descriptions from the teacher’s perspective corroborate the quantitative rating differences. In summary, for RQ2, both the teacher’s quantitative ratings and subjective observations confirm that affective scaffolding was associated with a higher level of child emotional engagement. This underscores the importance of teachers attending to and actively guiding students’ emotional states in the classroom: when a teacher provides positive emotional support, children’s emotional engagement increases, and the quality of classroom interaction and learning involvement rises accordingly (Pakarinen et al., 2020; Guo et al., 2025).

### 4.3 Differences in children’s visual attention patterns

Third, we found that affective scaffolding influenced children’s visual attention distribution. The eye-tracking results showed that in the affective condition, children devoted more attention to the Face AOI (areas of the story related to characters’ faces and expressions), while their attention to the Text AOI did not differ significantly from that of the instructional group. In other words, affective scaffolding guided children to focus more on social cues (such as facial expressions of characters) without sacrificing attention to the story text. This suggests a beneficial reallocation of attention: affective scaffolding additionally activated children’s attention to social–emotional cues, while not diminishing necessary attention to textual information (Park et al., 2020). In contrast, instructional scaffolding ensured children understood the plot and text, but the children’s social attention was not as fully engaged. Put differently, in the affective group, increased emotional engagement did not come at the expense of reading the text. Addressing RQ3, we conclude that affective scaffolding led children to exhibit a stronger social attention bias during reading—that is, they paid more attention to people (or characters) and their emotional cues (Kao et al., 2019)

This result can be interpreted from evolutionary and developmental psychology perspectives: social signals like facial expressions naturally attract children’s attention and have adaptive value (Elam et al., 2010). Affective scaffolding provided rich social cues (e.g., the teacher’s facial expressions, emotional tone), which effectively captured children’s attention and thus boosted their engagement (Elam et al., 2010). Building on social attention and social referencing frameworks, emotionally expressive teacher cues may operate as attentional signals that prioritize socially informative regions in the visual scene, prompting children to extract emotional meaning from characters’ faces during the narrative (Elam et al., 2010; Park et al., 2020; Ruba and Pollak, 2020). In contrast, a purely instructional, emotion-neutral approach is relatively dry and lacks those emotional signals, so children’s social attention mechanisms are not fully activated, and their engagement remains lower. From a sociocultural scaffolding lens, these affective cues (prosody, embodied emphasis, emotion-focused prompts) can be conceptualized as mediational tools that highlight “what matters” in the story, thereby shaping children’s moment-to-moment attention allocation during a shared activity—without reducing attention to text (Morcom, 2014; Veraksa et al., 2021). From an educational practice standpoint, this finding means that when teachers appropriately use facial expressions and body language—emotional cues—in teaching, they can effectively draw children’s attention, strengthen the emotional connection between teacher and students, facilitate information exchange, and promote children’s engagement (Park et al., 2020; Veraksa et al., 2021).

### 4.4 Differences in children’s behavioral engagement

Fourth, the behavior coding results showed that affective scaffolding significantly enhanced children’s overt engagement behaviors. In the affective group, children displayed more frequent positive interactive behaviors (Bailey et al., 2013; Sampson, 2025): behaviors such as pointing to pictures, using emotion-related words, retelling story content, and leaning forward occurred at markedly higher rates than in the instructional group. Conversely, for behaviors like asking for re-reading or showing distraction, there were no significant group differences. This means affective scaffolding stimulated more active exploration and participation behaviors from children, without reducing their need for comprehension or completely eliminating lapses in attention (Chen and Adams, 2023). The Behavioral Engagement Index (BEI), computed from multiple behavior indicators, was significantly higher in the affective group than the instructional group, further demonstrating that emotional scaffolding effectively raised children’s overall level of behavioral participation.

From a motivational standpoint, affective scaffolding created a pleasant and meaningful learning experience, which sparked children’s inclination to explore and participate actively (Veraksa et al., 2021; Xiao et al., 2025). When children feel that the learning process is enjoyable and meaningful, they are more likely to spontaneously point out, comment on, and reenact story content. Research has noted that students’ emotional experiences and behavioral engagement often go hand-in-hand—positive emotional states translate into more sustained effort and concentration (Skinner and Belmont, 1993). Our findings are consistent with this view: the positive emotions induced by affective scaffolding led children to exhibit more proactive behaviors (such as asking to revisit parts of the story, pointing things out, or even role-playing lines), forming a virtuous cycle of emotion–behavior interaction (Bailey et al., 2013).

Notably, the lack of group differences in re-reading requests and distraction suggests that affective scaffolding selectively amplified engagement-relevant behaviors (e.g., emotion talk, pointing, retelling, proximity) rather than indiscriminately increasing all observable actions. This selectivity strengthens the interpretation that affective scaffolding supports children’s agentic participation instead of merely producing generalized activity or restlessness (Skinner and Belmont, 1993; Chen and Adams, 2023).

From a sociocultural theory perspective, this high level of engagement suggests that children were participating as agents in a shared activity (Renshaw, 2013; Morcom, 2014). By providing affective scaffolding, the teacher essentially built a platform where children felt safe and encouraged to immerse themselves and explore, and the children in turn demonstrated greater initiative and agency on that platform (Colombetti and Krueger, 2015). In ZPD terms, the teacher’s affective cues and empathic responses may have lowered the social–emotional “entry cost” of participation, enabling children to contribute more spontaneously (e.g., pointing and retelling) and to externalize emotional meaning through language (emotion words) (van de Pol et al., 2010; Colombetti and Krueger, 2015). Qualitative data also underscored this: children in the affective group showed more self-initiated behaviors and spontaneous interactions, such as volunteering questions or asking to take part in demonstrations, indicating an increase in their learning agency. In contrast, under instructional scaffolding, children were more often passively answering questions, with fewer emotionally driven improvised interactions. Summarizing for RQ4, affective scaffolding significantly enhanced a broad range of children’s active engagement behaviors, indicating that infusing teaching with emotional elements can be converted into tangible participatory actions (Wilson and Gottman, 1996; Ho, 2025).

### 4.5 Consistency across multiple indicators and mechanisms of emotional engagement

Lastly, our study examined the consistency among different engagement dimensions and how they relate to overall engagement. The correlation analysis showed that children’s SAM valence, SAM arousal, teacher engagement ratings, face-gaze proportion, and BEI were all positively interrelated at moderate levels. This result is important: it indicates that a child’s emotional experience, attention distribution, overt behavior, and teacher-observed engagement are interlinked, pointing to a common underlying construct—namely, the child’s emotional engagement level (Wenren et al., 2024; Ho, 2025). In other words, when a child feels happy and excited, they also tend to visually focus more intently, participate more actively, and are rated as more engaged by the teacher (Gatcho et al., 2024). These inter-correlations among multiple indicators provide convergent evidence for the study’s conclusions.

Previous literature conceptualizes student classroom engagement as a multi-dimensional composite in which emotional and behavioral dimensions are distinct yet related (Renshaw, 2013; Torto, 2020; Seah and Coifman, 2024). Our data support this view: children with high emotional engagement also generally displayed high behavioral engagement, with the two reinforcing each other. Meanwhile, the moderate correlation coefficients also indicate that these measures are not identical—each captures something unique. This suggests that “emotional engagement” is a complex, multi-faceted concept. However, the overall pattern indicates that under affective scaffolding, these dimensions rose in step and in harmony. Qualitative data similarly reflected this consistency: in affective scaffolding classrooms, teachers described children as “intensely focused, frequently nodding and smiling,” and children reported “it was really fun and made me happy,” even proactively asking, “Can I answer this question?” Such on-site observations and post-activity feedback mutually corroborate each other, indicating that the impact of affective scaffolding was comprehensive rather than confined to any single aspect (Morcom, 2014; Seah and Coifman, 2024).

Crucially, this cross-method coherence is theoretically informative: it is consistent with an integrated mechanism in which affective scaffolding simultaneously (a) shapes children’s emotional experience via warmth and empathic affirmation (motivation-related processes), (b) orients attention toward socially informative cues (social attention processes), and (c) supports agentic participation within a shared meaning-making activity (sociocultural scaffolding processes). In this interpretation, emotion, attention, and behavior are not parallel outcomes but mutually reinforcing components of engagement that are coordinated through the teacher’s affective guidance during reading (Park et al., 2020; Wenren et al., 2024; Ho, 2025).

In answer to RQ5, our conclusion is that the improvements in children’s emotional experience, attention, and engagement behaviors brought about by affective scaffolding were consistent and mutually supporting, exemplifying the integrated concept of “emotional engagement.” In educational practice, children’s emotional, cognitive, and behavioral engagement are interwoven; therefore, simultaneously examining multiple dimensions to fully understand student engagement is necessary. Our study provides an example: by combining subjective self-reports, teacher assessments, objective eye-tracking, and behavior observation, we captured a more comprehensive measure of engagement and showed that with ample emotional support these facets improve in tandem.

### 4.6 Practical implications

Our findings offer several implications for educational practice. First, they remind teachers and educators to prioritize emotional factors in the classroom. In teaching, a teacher is not only a transmitter of knowledge but also a “regulator” and model of students’ emotions. The success of affective scaffolding in this study shows that when teachers invest positive emotional attitude in teaching, they can effectively infect and energize students (Colombetti and Krueger, 2015; Veraksa et al., 2021). For example, if teachers tell stories or conduct lessons with genuine enthusiasm and rich expressions, and openly share emotional reactions to the content (surprise, joy, sympathy, etc.), students are often swept up in the atmosphere and become more deeply engaged in learning. Therefore, teachers should consciously integrate emotion education strategies into lesson planning and classroom interaction—for instance, using storytelling, analogies, or questions to prompt students to consider characters’ feelings; giving timely acknowledgment and response to students’ emotional reactions; and fostering a warm, safe classroom climate that encourages children to express their feelings (Sampson, 2025). In frontline teacher training, the cultivation of emotional support skills should be emphasized. This includes learning to detect students’ emotional needs, using tone of voice and body language to convey care and encouragement, and finding emotional elements in academic content (e.g., discussing the feelings in a story or historical event to guide student empathy) (Colombetti and Krueger, 2015; Chen and Adams, 2023). Empirical research has shown that the level of teacher emotional support is closely associated with students’ engagement and academic performance (Guo et al., 2025). Therefore, in teacher professional development, emotional instructional skills should be valued on par with academic teaching skills.

Beyond individual teachers’ efforts, schools can also take steps: incorporate social–emotional learning into the curriculum, using class meetings or group activities to cultivate students’ ability to recognize and express emotions and to foster a positive emotional climate in the classroom (Renshaw, 2013); include observation of teacher emotional support as a criterion when evaluating teaching quality, to encourage teachers to build “positive emotion classrooms” (Park et al., 2020). Moreover, our study provides evidence supporting the notion that emotion education is not at odds with academic learning—rather, the two can complement each other. By enhancing students’ engagement through emotional involvement, learning and comprehension can be deepened. This suggests that infusing curricula with emotional elements (such as role-play, situational experiences, discussions of feelings) may improve learning outcomes (Sampson, 2025). This is especially relevant for preschool and early elementary children, whose learning heavily relies on emotional attachment to the teacher and their affective evaluation of activities (Colombetti and Krueger, 2015). Implementing instruction characterized by emotional support at these stages can provide better learning experiences for children and ignite their long-term enthusiasm for learning (Park, 2016). For parents and tutors outside of school, the concept of affective scaffolding is also instructive—when supporting a child’s learning, providing abundant positive emotional feedback and empathic support (rather than only checking answers or giving commands) can make the child’s experience more enjoyable and encourage greater independent participation and persistence (Renshaw, 2013). In sum, the practical value of affective scaffolding lies in reminding us that good education involves not just “teaching content” but also “nurturing the person,” including cultivating students’ positive and healthy emotional experiences. A simple smile, a word of praise, or an emotionally rich story in the classroom can often ignite a child’s spark for learning. These humanizing details of teaching should become standard practice for educators, rather than optional embellishments.

### 4.7 Limitations and future directions

This study has several limitations. First, the sample size was modest and drawn from a single region’s preschool, which may limit the generalizability of the findings. Future research could replicate this study in different cultural and educational settings to test the universality of affective scaffolding effects. Second, our quasi-experimental design captured primarily immediate effects in a single session; we did not examine the longer-term influence of affective scaffolding on children’s sustained motivation or emotional development. Follow-up longitudinal or intervention studies are needed to explore the stability and extended impact of affective scaffolding over time. Third, we did not consider individual difference variables such as children’s emotional temperament or teachers’ personal emotional expressiveness, which might moderate the effectiveness of affective scaffolding. Future studies could include measures of children’s emotion regulation abilities or teachers’ socio-emotional skills to enrich the analysis of underlying mechanisms. Fourth, in statistics, we acknowledge that a small sample size limits statistical power, and for comparisons with smaller effect sizes, we cannot rule out the possibility of false positives or false negatives. These exploratory findings provide hypotheses for future research but require independent validation with larger samples. Lastly, our study focused on the engagement process and did not directly assess learning outcomes. Future research could incorporate comprehension tests or transfer tasks to further verify whether affective scaffolding also enhances cognitive learning results. In general, future studies should expand to larger samples, use longitudinal perspectives, and integrate multi-level variables to continue exploring how affective scaffolding influences children’s socio-emotional development and learning trajectories.

## 5 Conclusion

Addressing five specific questions, this study investigated the role of affective scaffolding in preschoolers’ learning, using mixed quantitative and qualitative data to arrive at the following main conclusions: Compared to traditional instructional scaffolding, affective scaffolding significantly improved children’s emotional experience, increasing their positive affect and physiological arousal levels; teachers also more frequently observed overt emotional engagement in children, and the classroom emotional atmosphere was richer. Under affective scaffolding, children directed more attention to social cues (such as the teacher’s or characters’ facial expressions) without any decrease in attention to textual content; children showed stronger behavioral engagement, with more proactive interactive behaviors, reflecting a higher level of participatory learning; and the indicators of emotion, attention, behavior, and engagement converged, all showing consistent positive changes, indicating that the impact of affective scaffolding was broad and coordinated. These findings underscore the practical value of “emotion” in teaching—emotion is not a mere accessory to learning, but indeed one of its driving forces for deeper engagement. Theoretically, our research enriches the concept of scaffolding by incorporating emotional support into the framework of effective teaching strategies, aligning with sociocultural theory’s emphasis on supportive emotional environments. The evidence from this study shows that integrating emotional factors into instructional design and classroom interaction can elicit higher engagement and participation from young children, which has positive implications for fostering children’s interest in learning, developing good teacher–child relationships, and supporting long-term academic growth. Educators should value and skillfully employ emotion as an “invisible curriculum,” using genuine care and enthusiastic guidance to make the classroom a place where children want to learn and enjoy learning. As research in this area deepens, we expect to further unveil the far-reaching effects of affective scaffolding on learning motivation, cognitive development, and socio-emotional skills, providing even more insights for educational practice.

## Data Availability

The raw data supporting the conclusions of this article will be made available by the authors, without undue reservation.
