# Supplementary material for: Effects of affective vs. instructional teacher scaffolding on preschoolers’ emotional engagement and social attention in picturebook reading
Source: Front Psychol. 2026 Feb 10;17:1743106. doi: 10.3389/fpsyg.2026.1743106 (PMC12929952; doi:10.3389/fpsyg.2026.1743106)
Supplement: Supplementary file 1 [file Data_Sheet_1.PDF]

Semi-Structured Interview Guide for Children

Purpose: To explore children's emotional responses, empathic engagement, and sense of involvement during the picturebook reading activity.

1. Which page did you like the most? Why?
2. Was there any page that made you feel scared or uncomfortable? Why?
3. If you were on the boat, what would you do?
4. How do you think the little duck felt during the storm?
5. Did you feel happy at the end of the story? Why?
6. When the teacher was reading, did her voice or facial expression feel special to you? Did you enjoy it?
7. While reading, did you want to tell the teacher or your friends about your feelings?
8. What do you think about the part where the duck's friends helped each other?

Semi-Structured Interview Guide for Teachers

Purpose: To understand how teachers guided children's emotional experiences during the reading, observed children's reactions, and reflected on the scaffolding approach.

1. How did you use language or tone to help children understand emotions?
2. During which parts of the story did the children seem most engaged, and how did you respond?
3. Did you observe any children showing clear emotional reactions? Could you provide examples?
4. What kinds of emotional expressions did the children show during the reading? Were they different from usual?
5. Compared to your regular story sessions, did your approach to guidance differ during this activity? Please describe.
6. In your opinion, what are the differences between the affective and instructional guiding approaches?
7. In your opinion, how does emotional education play a role in your teaching?
8. If you were to continue using the affective scaffolding approach in future sessions, what aspects would you like to improve or enhance?

Implementation Fidelity Checklist for Scaffolding Styles

Purpose

This checklist was used to verify whether teachers consistently implemented the assigned scaffolding style (affective vs. instructional) during the electronic picturebook reading sessions.

Rating Procedure

Each reading session was independently reviewed by two trained researchers based on video recordings. Items were rated as **Present (1)** or **Absent (0)**. Sessions were considered to meet fidelity criteria only when the majority of indicators corresponding to the assigned condition were observed and no systematic crossover between conditions occurred.

A. Affective Scaffolding Indicators

No.	Indicator	Description
A1	Use of emotion-focused prompts	Teacher explicitly invited children to reflect on characters' feelings (e.g., "How do you think the little duck feels here?").
A2	Empathic verbal responses	Teacher acknowledged and validated children's emotional expressions (e.g., "Yes, that does sound scary.").
A3	Expressive prosody	Teacher used varied tone, pitch, and rhythm to convey emotional meaning during narration.
A4	Facial expressiveness	Teacher displayed clear facial expressions aligned with the emotional content of the story.
A5	Embodied emotional cues	Teacher used gestures or body movements to emphasize emotional moments (e.g., leaning forward, widening eyes).
A6	Encouragement of emotional sharing	Teacher encouraged children to share their own feelings or personal reactions to the story.
A7	Warm and supportive interaction style	Overall interaction conveyed warmth, responsiveness, and emotional attunement.

B. Instructional Scaffolding Indicators

No.	Indicator	Description
I1	Use of factual or plot-based questions	Teacher focused on identifying characters, actions, or sequence (e.g., "Who is this?" "What happened next?").
I2	Neutral instructional tone	Teacher maintained a neutral, matter-of-fact tone without expressive emotional modulation.
I3	Emphasis on comprehension accuracy	Teacher prioritized correct answers and story understanding over emotional reflection.
I4	Limited emotional	Teacher did not expand on or probe children's

	elaboration	emotional responses.
I5	Directive questioning style	Teacher-led interaction with predetermined questions guiding children's responses.
I6	Minimal use of embodied emotional cues	Little to no use of gestures or facial expressions to convey emotional meaning.

C. Fidelity Decision Criteria

A session was judged as **high-fidelity** if:

For the **affective scaffolding condition**, the majority of indicators A1–A7 were present, and instructional indicators (I1–I6) did not dominate the interaction.

For the **instructional scaffolding condition**, the majority of indicators I1–I6 were present, with minimal use of affective indicators (A1–A7).

No systematic blending of the two scaffolding styles was observed.

Disagreements between raters were resolved through discussion until consensus was reached. No sessions were excluded due to insufficient fidelity.
